# Perioperative immunotherapy in gastric cancer: walking a fine line between hope and caution

**DOI:** 10.3389/fimmu.2025.1722749

**Published:** 2025-12-17

**Authors:** Xianlei Cai, Xia Xiao, Congcong Zhang, Weiming Yu

**Affiliations:** Department of Gastrointestinal Surgery, The Affiliated Lihuili Hospital of Ningbo University (Ningbo Medical Center Lihuili Hospital), Ningbo, Zhejiang, China

**Keywords:** perioperative immunotherapy, gastric cancer, clinical trial, biomarkers, pathological response rate

## Abstract

Perioperative immunotherapy has emerged as an important strategy in the management of resectable gastric and gastroesophageal junction adenocarcinoma. Phase II and III studies combining immune checkpoint inhibitors with chemotherapy have shown higher pathological response rates and improvements in event-free outcomes, particularly in molecularly selected groups such as HER2-positive and MSI-H or dMMR tumors. MSI-H and dMMR cancers show marked sensitivity to immune treatment, often achieving high rates of pathological complete response. Combinations that include HER2-directed therapy and immunotherapy have also produced encouraging antitumor activity. However, the results in broader, unselected populations remain variable, and reliable predictive markers such as PD-L1 are still lacking. While safety profiles are generally acceptable, some treatment regimens, especially those involving antiangiogenic agents or dual checkpoint blockade, call for careful perioperative evaluation. Importantly, despite improvements in pathological and early clinical outcomes, the impact on overall survival has been limited so far, and longer follow-up is needed to clarify the true survival benefit. Future progress will depend on better patient selection through integrated molecular and immune markers, more thoughtful sequencing of therapies, and the development of combination strategies that can enhance the durability of response. These findings highlight both the promise of perioperative immunotherapy and the need for continued efforts to achieve meaningful survival gains.

## Introduction

Gastric cancer remains a significant global health burden (1), with conventional perioperative chemotherapy regimens such as FLOT (fluorouracil, leucovorin, oxaliplatin, and docetaxel) demonstrating limited efficacy in preventing recurrence (2). Immunotherapy has fundamentally reshaped the therapeutic landscape for advanced gastric cancer, establishing a new standard of care in the metastatic setting (3–5). The integration of immune checkpoint inhibitors (ICIs) into perioperative treatment strategies has emerged as a promising approach to improve outcomes for patients with resectable gastric and gastroesophageal junction (G/GEJ) adenocarcinoma. This paradigm shift is supported by robust evidence from metastatic settings, where immunotherapy has substantially improved survival (6). The unique immunological microenvironment of gastric cancer, particularly in subtypes with microsatellite instability-high (MSI-H) or programmed death-ligand 1 (PD-L1) positivity, provides a strong rationale for incorporating ICIs in earlier disease stages. This review synthesizes recent advancements from key phase II and III clinical trials investigating perioperative immunotherapy, highlighting efficacy endpoints, biomarker exploration, and safety profiles (**Table 1**). Furthermore, it examines specialized approaches for HER2-positive and dMMR/MSI-H subgroups, discusses predictive biomarkers, and outlines future research directions aimed at personalizing treatment and overcoming resistance mechanisms.

**Table 1:** Characteristics of key phase II and III neoadjuvant/perioperative immunotherapy ± chemotherapy trials in resectable gastric and gastroesophageal junction cancer.

Year	Study name/identifier	Phase	Experimental/control arm (n)	Experimental regimen	Control regimen	MPR rate (%)	pCR rate (%)	R0 (%)	EFS/DFS/PFS	OS	Grade 3–4 adverse events (%)
Unselected											
2025	MATTERHORN	Phase III, double-blind, randomized, international	533/530	Durvalumab + FLOT	Placebo + FLOT	33 vs 24	19 vs 10	93 vs 93	24-mo EFS: 67.4% vs 58.5%, HR:0.71; 95%CI: (0.58-0.86)	3-year OS: 68.6% vs 61.9% HR:0.78; 95%CI: (0.63-0.95)	71.6 vs 71.2
2024	KEYNOTE-585	Phase III, randomized, double-blind, multicenter	503/498 (Cohort D: pemb+ FP); 280/279 (Cohort F: pemb+ CAPOX)	Pemb + chemo (FP or CAPOX)	Placebo + chemo (FP or CAPOX)	28 vs 15 (FP cohort); 24 vs 12 (CAPOX cohort)	13 vs 5 (FP); 10 vs 3 (CAPOX)	91 vs 90	mEFS 44.4m vs 25.7m; HR:0.81; 95%CI: (0.67-0.98)	mOS 71.8m vs 55.7m; HR:0.86; 95%CI: (0.71-1.06)	71 vs 68
2025	DRAGON IV/CAP-05	Phase III, randomized, multicenter	180/180	Camrelizumab + rivoceranib + SOX (SOXRC))	SOX	51.1 vs 37.8	18.3 vs 5.0	99 VS 94	N/A	N/A	34 vs 17
2025	MONEO	Phase II	40	Avelumab + FLOT4:	None	28.9	21.1%	N/A	3-year DFS: 66%	3-year OS: 69%	80
2024	N/A	Phase II	32	Sintilimab + FLOT	None	55.2	17.2	93.1	3-year EFS: 71.4	3-year OS: 70.9	59.4
2024	NEOSUMMIT-01	Phase II, randomized	54/54	Toripalimab + SOX/XELOX	SOX/XELOX	44.4 vs 20.4	22.2 vs 7.4	N/A	N/A	N/A	35.2 vs 29.6
2025	NEOSUMMIT-03	Phase II	32	Tislelizumab + SOX	None	50.0	28.1	96.9	N/A	N/A	31.2
2024	NCT04195828	Phase II, multicenter, randomized	51/53	Camrelizumab + Apatinib + nab-Paclitaxel + S-1	nab-Paclitaxel + S-1	33.3 VS 17.0	N/A	94.1	N/A	N/A	33.3 vs 26.4
2022	Neo-PLANET	Phase II	36	Camrelizumab + concurrent chemoradiotherapy	None	44.4	33.3	91.7	2-year PFS: 66.9%	2-year OS: 76.1%	75.0
2024	PANDA	Phase II	21	Atezolizumab + docetaxel + oxaliplatin + capecitabine	None	70	45	100	N/A	N/A	10
2025	NCT03288350	Phase II	51	Avelumab+ docetaxel + cisplatin + 5-fluorouracil	None	18	14	N/A	2-year DFS: 67.5%	N/A	40
2024	WuhanUHGI001	Phase II	49	Tislelizumab + SOX	None	49.0	26.5	N/A	2-year PFS: 69.4%	2-year OS: 81.2%	12.2
2024	NCT05602935	Phase II	29	Camrelizumab + SOX	None	69.0	10.3	96.6	N/A	N/A	0
Her2+											
2025	ChiCTR2200058732	Phase II	22	Sintilimab + Trastuzumab)+ chemotherapy	None	55	50	N/A	N/A	N/A	27
2025	NCT04661150.	Phase II, randomized	21/21	Atezolizumab + Trastuzumab + XELOX	Trastuzumab + XELOX	N/A	38 VS 14	N/A	N/A	N/A	57 VS 67
2025	NCT03950271	Phase II	25	Camrelizumab + Trastuzumab + CapOx	None	30.4	21.7	100	3-year DFS: 78.3	N/A	36
MSI-H/dMMR											
2023	NEONIPIGA	Phase II	32	Nivolumab + Ipilimumab	None	N/A	58.6	100	N/A	N/A	19
2025	INFINITY	Phase II	18	Tremelimumab + Durvalumab	None	80	60	N/A	2-year PFS: 85	2-year OS: 92	17
2025	NICE	Phase II	16	Toripalimab + CapeOX	None	93.3	80	100	N/A	N/A	37.5

## Perioperative immunotherapy combined with chemotherapy in gastric cancer: phase III clinical research progress

The phase III MATTERHORN trial represents a landmark study evaluating the addition of durvalumab to FLOT chemotherapy in resectable G/GEJ adenocarcinoma (7). This multinational, double-blind, randomized trial demonstrated a significant improvement in event-free survival (EFS) with durvalumab plus FLOT compared to FLOT alone (67.4% vs. 58.5% at 2 years; HR 0.71). The pathological complete response (pCR) rate was substantially higher in the durvalumab group (19.2% vs. 7.2%), and overall survival (OS) also favored the experimental arm (75.7% vs. 70.4% at 2 years) (7). These findings establish a new benchmark for perioperative immunotherapy in unselected populations.

Similarly, the KEYNOTE-585 trial investigated pembrolizumab combined with cisplatin-based chemotherapy or FLOT (8). While it met its primary endpoint for pCR (12.9% vs. 2.0%), the improvement in EFS did not reach statistical significance (HR 0.81), suggesting that not all immunotherapy combinations yield equivalent benefits (8). The contrasting outcomes between MATTERHORN and KEYNOTE-585 may be attributed to differences in chemotherapy backbones, patient selection, or immunotherapy agents.

The DRAGON IV/CAP 05 trial explored a novel combination of camrelizumab (anti-PD-1), rivoceranib (VEGFR-2 inhibitor), and chemotherapy (SOX) (9). This regimen significantly improved pCR rates compared to chemotherapy alone (18.3% vs. 5.0%), indicating that dual targeting of PD-1 and VEGF pathways may provide synergistic antitumor effects (9).

For adjuvant therapy specifically, the ATTRACTION-5 trial evaluated nivolumab plus chemotherapy after D2 gastrectomy in stage III gastric cancer (10). Contrary to expectations, the addition of nivolumab did not improve relapse-free survival (HR 0.90), highlighting the potential differences between neoadjuvant and adjuvant immunotherapy approaches (10). This suggests that the immunosuppressive microenvironment following surgery may limit the efficacy of adjuvant immunotherapy, or that patient selection criteria need refinement.

These phase III trials collectively demonstrate that while perioperative immunotherapy shows promise, its benefits are not uniform across all regimens and patient populations. The MATTERHORN regimen has emerged as a potential new standard of care, while other combinations require further optimization.

## Perioperative immunotherapy combined with chemotherapy in gastric cancer: phase II clinical research progress

Phase II trials have been instrumental in exploring diverse immunotherapy combinations and identifying responsive patient subsets. The MONEO trial evaluated avelumab plus FLOT, demonstrating a pCR rate of 21.1% and a major pathological response (MPR) rate of 28.9% (11). Importantly, responses were more pronounced in patients with PD-L1 combined positive score (CPS) ≥10 (33.3% vs. 21.1%), providing early evidence for biomarker-driven patient selection (11).

Several studies have investigated PD-1 inhibitors with chemotherapy backbones commonly used in Asian populations. A phase II trial of sintilimab plus FLOT in HER2-negative G/GEJ cancer showed a pCR rate of 17.2% and MPR rate of 55.2% (12). The NEOSUMMIT-01 trial demonstrated that toripalimab plus SOX/XELOX significantly improved tumor regression grade (TRG) 0/1 rates compared to chemotherapy alone (44.4% vs. 20.4%) (13). Similarly, tislelizumab plus SOX in the NEOSUMMIT-03 trial achieved an MPR rate of 50.0% and pCR rate of 25.0% (14).

The combination of immunotherapy with antiangiogenic agents has also shown promise. A randomized phase II trial found that camrelizumab and apatinib combined with chemotherapy (nab-paclitaxel plus S-1) significantly improved MPR rates compared to chemotherapy alone (33.3% vs. 17.0%) (15). This approach targets multiple pathways simultaneously and may benefit patients with immunosuppressive tumor microenvironments.

For radiotherapy combinations, the Neo-PLANET trial evaluated camrelizumab with concurrent chemoradiotherapy, demonstrating a pCR rate of 33.3% (16). This suggests that radiotherapy may enhance the immunogenicity of tumors and synergize with immunotherapy, though this approach requires further validation.

These phase II trials collectively indicate that perioperative immunotherapy combinations can achieve pCR rates of 15-30% and MPR rates of 30-55% in unselected populations. The variability in responses highlights the need for better patient selection strategies and the importance of biomarker development.

## HER2-positive gastric cancer: perioperative immunotherapy combined with targeted therapy

HER2-positive gastric cancer represents a distinct subtype where dual blockade of HER2 and immune checkpoints may yield synergistic effects. A phase II trial evaluating sintilimab (PD-1 inhibitor) plus trastuzumab and chemotherapy demonstrated impressive results, with 55% of patients achieving MPR and 50% achieving pCR (17). These response rates substantially exceed those historically observed with trastuzumab and chemotherapy alone, suggesting that immune activation may enhance the antitumor effects of HER2 blockade.

Similarly, a randomized phase II trial compared atezolizumab plus trastuzumab and XELOX versus trastuzumab and XELOX alone (18). The addition of atezolizumab significantly improved pCR rates (38% vs. 14%), particularly in patients younger than 65 years, males, and those with intestinal-type histology (18). This study provides further evidence that immune checkpoint inhibition can augment the efficacy of HER2-targeted therapy.

The single-arm phase II trial of camrelizumab plus trastuzumab and CapOx showed a pCR rate of 21.7% and near-pCR rate of 30.4% (19). Importantly, no patients achieving pCR experienced recurrence during follow-up, suggesting that pathological response may serve as a surrogate for long-term outcomes in this population (19).

Mechanistic studies have revealed that HER2-targeted therapy may enhance antigen presentation and T-cell infiltration, potentially creating a more favorable microenvironment for immunotherapy (17, 18). However, resistance mechanisms involving regulatory T cells have been identified, suggesting that additional immunomodulatory strategies may be needed for complete responses (17).

These findings position immunotherapy plus HER2-targeted therapy as a promising approach for HER2-positive gastric cancer, with several regimens demonstrating superior pathological response rates compared to historical controls. Ongoing trials are further exploring this combination and seeking to identify optimal patient selection criteria.

## dMMR/MSI-H gastric cancer: perioperative immunotherapy and non-operative management

Deficient mismatch repair (dMMR) or microsatellite instability-high (MSI-H) gastric cancers exhibit exceptional sensitivity to immune checkpoint blockade due to their high tumor mutational burden and abundant neoantigen presentation. The NEONIPIGA phase II study evaluated neoadjuvant nivolumab plus ipilimumab (CTLA-4 inhibitor) in locally advanced resectable dMMR/MSI-H G/GEJ adenocarcinoma (20). This chemo-free regimen achieved a remarkable pCR rate of 58.6% among patients who underwent surgery, demonstrating that immunotherapy alone can induce substantial tumor regression in this molecular subset (20).

The INFINITY study took a more ambitious approach by investigating non-operative management for dMMR/MSI-H tumors achieving clinical complete response (cCR) after tremelimumab plus durvalumab (21). In cohort 1 (neoadjuvant), the pCR rate was 60%, while in cohort 2 (non-operative management), 13 of 17 assessable patients achieved cCR and avoided surgery (21). With a median follow-up of 11.5 months, the 12-month gastrectomy-free survival was 64.2%, providing preliminary evidence that selected patients may be spared surgical morbidity without compromising outcomes (21).

The NICE trial reported exceptional pathological complete response rates of 80% in dMMR/MSI-H patients treated with neoadjuvant toripalimab plus CapeOX, yet such dramatic responses in molecularly enriched populations may not reflect outcomes in broader patient groups (22).

These findings challenge the traditional paradigm of mandatory surgical resection for localized gastric cancer and suggest that organ preservation may be feasible in dMMR/MSI-H patients achieving complete response to immunotherapy (20, 21). However, longer follow-up is needed to confirm the durability of these responses and establish reliable criteria for patient selection. The exceptional response of dMMR/MSI-H tumors to immunotherapy underscores the importance of universal molecular testing in gastric cancer patients. These results also provide a strong rationale for upfront immunotherapy in this population, potentially avoiding the toxicity of chemotherapy while achieving superior outcomes.

## Biomarkers and efficacy prediction

Identifying reliable biomarkers to predict response to perioperative immunotherapy remains a critical research focus. PD-L1 expression, as measured by combined positive score (CPS), has shown variable predictive value across studies (11, 23). While the MONEO trial observed better responses in patients with CPS ≥10 (11), other studies found no correlation between PD-L1 status and pathological response (12, 24), suggesting limited utility as a standalone biomarker.

MSI-H/dMMR status consistently predicts exceptional response to immunotherapy (20, 21)., though this molecular subtype represents only a small proportion of gastric cancers. Beyond MSI status, multi-omics approaches have identified several potential predictive biomarkers. Transcriptomic analyses have revealed that regulatory T cell infiltration is associated with resistance to dual PD-1/HER2 blockade (17), while M2-tumor-associated macrophage proliferation correlates with lack of response (24).

A prospective phase II trial identified intestinal subtype by Lauren classification as a key predictor of sensitivity to neoadjuvant immunochemotherapy (25). Mechanistically, intestinal-type tumors exhibited increased DNA damage repair-active cancer cells and enrichment of CLEC9A+ dendritic cells in the tumor microenvironment (25). Based on these findings, a machine learning model integrating transcriptomic features of both epithelial and immune compartments was developed to accurately predict treatment response (25).

Peripheral immune markers have also shown promise. The WuhanUHGI001 trial found that preoperative circulating tumor cells combined with pathological responses helped in prognosis assessment (26). Additionally, T downstaging, lymphocyte-to-monocyte ratio, and CD3+ T cells were independent factors affecting progression-free survival (26).

Emerging biomarkers beyond conventional markers show promise in refining patient selection. Tumor microenvironment characteristics, including the presence of PDPN+ cancer-associated fibroblasts, have been associated with reduced likelihood of pathological response to immunotherapy (27). Similarly, CD8A expression and PGF levels have demonstrated predictive value for treatment response in gastric cancer patients receiving neoadjuvant immunotherapy (28). Multimodal biomarker approaches incorporating circulating tumor DNA, immune cell infiltration patterns, and transcriptomic signatures may further enhance patient selection (26, 29). The development of validated predictive models integrating clinical, pathological, and molecular variables represents an essential step toward personalized perioperative immunotherapy (26).

These emerging biomarkers reflect the complex interplay between tumor cells and the immune microenvironment. While no single biomarker has yet been validated for routine clinical use, multi-parameter models incorporating tumor and immune characteristics show promise for personalizing perioperative immunotherapy approaches.

## Safety management and treatment-related adverse events

The safety profile of perioperative immunotherapy combinations is generally manageable, with most adverse events being attributable to the chemotherapy component. In the phase III MATTERHORN trial, the incidence of grade 3-4 adverse events was similar between the durvalumab and placebo groups (71.6% vs. 71.2%) (7). Similarly, the KEYNOTE-585 trial reported comparable rates of grade 3 or higher adverse events between pembrolizumab and placebo arms (78% vs. 74%) (8).

Common immunotherapy-related adverse events include immune-mediated toxicities such as rash, thyroid dysfunction, and hepatitis, which are generally manageable with corticosteroids and treatment interruption (19, 30). The incidence of these events varies across studies, with some reporting grade 3-4 immune-related adverse events in approximately 10% of patients (23).

Surgical safety is a particular concern in the perioperative setting. Most trials have reported no significant increase in surgical complications or mortality with the addition of immunotherapy (7, 13, 14). For instance, the NEOSUMMIT-01 trial found comparable surgical morbidity between toripalimab plus chemotherapy and chemotherapy alone groups (11.8% vs. 13.5%) (13).

However, specific combinations may present unique safety considerations. The integration of immunotherapy into perioperative treatment protocols introduces unique safety considerations that extend beyond those observed in metastatic disease. Patients undergoing multimodality therapy for locally advanced gastric cancer face compounded toxicities from combined surgical stress, chemotherapy, and immune activation (31). The addition of antiangiogenic agents to immunotherapy and chemotherapy can increase the risk of hypertension, proteinuria, and wound healing complications (9, 15). Immune-related adverse events, including myocarditis, hepatitis, pneumonitis, and rare manifestations such as ureteritis/cystitis, present particular challenges in the preoperative period, potentially delaying curative surgery or complicating postoperative recovery (32, 33).

The perioperative period represents a physiologically vulnerable state characterized by surgical stress, transient immunosuppression, and metabolic alterations that may modulate both the efficacy and toxicity of immunotherapy (29, 34). This altered immune context raises legitimate concerns about potentially exacerbating irAEs during surgical recovery (32). Furthermore, the VESTIGE trial demonstrated that adjuvant nivolumab/ipilimumab actually resulted in worse disease-free survival compared to chemotherapy in high-risk patients (ypN+ and/or R1) following neoadjuvant chemotherapy and resection, highlighting that immune interventions are not universally beneficial even in settings of minimal residual disease (33). This sobering result emphasizes that the perioperative immune environment may differ fundamentally from that of advanced disease, necessitating careful risk-benefit assessment for each patient.

These findings highlight the importance of careful patient selection, proactive monitoring, and multidisciplinary management when implementing perioperative immunotherapy. While the toxicity profile is generally acceptable, specific combinations may require additional precautions and specialized management approaches.

## Conclusion and future perspectives

Perioperative immunotherapy has fundamentally transformed the treatment landscape for resectable gastric cancer, with phase III trials demonstrating improved pathological responses and survival outcomes (7, 9). The integration of immune checkpoint inhibitors with chemotherapy has established a new standard of care, particularly for microsatellite stable tumors (7). For molecularly selected populations, including HER2-positive and dMMR/MSI-H subgroups, targeted combinations and chemo-free approaches have shown remarkable efficacy.

Current evidence shows that perioperative immunotherapy can improve pCR and EFS in patients with advanced gastric cancer, but the overall survival benefit remains limited. Because OS is the most meaningful outcome for patients, these earlier improvements should be viewed with caution. Several reasons may explain the modest impact on OS, including relatively short follow-up, the biological diversity of gastric cancer, the use of additional treatments after recurrence, and the fact that a better pathological response does not always translate into long-term survival gains.

Future research should focus on several key areas. First, optimizing patient selection through validated biomarkers remains paramount. While PD-L1 and MSI status provide some guidance, more comprehensive biomarkers incorporating tumor and immune microenvironment characteristics are needed. Second, the sequencing and duration of perioperative therapy require refinement, particularly regarding the role of adjuvant immunotherapy after achieving pathological complete response. Third, novel combinations targeting complementary pathways, such as VEGF or LAG-3, may benefit patient subsets with inherent resistance to PD-1/PD-L1 blockade (15, 35).

The exploration of non-operative management for exceptional responders represents another frontier (21). While preliminary data from the INFINITY study are encouraging, longer follow-up and larger cohorts are needed to validate this approach (21). Finally, understanding and overcoming resistance mechanisms through translational research will be crucial for further improving outcomes (17).

In conclusion, perioperative immunotherapy has ushered in a new era for gastric cancer treatment, offering unprecedented response rates and survival benefits for selected patients. Through continued research and biomarker development, these approaches will become increasingly personalized and effective, ultimately improving outcomes for patients with this challenging disease.
